# Clinical characteristics and treatment outcomes in six cases of malignant tenosynovial giant cell tumor: initial experience of molecularly targeted therapy

**DOI:** 10.1186/s12885-018-5188-6

**Published:** 2018-12-29

**Authors:** Robert Nakayama, Jyothi Priya Jagannathan, Nikhil Ramaiya, Marco L. Ferrone, Chandrajit P. Raut, John E. Ready, Jason L. Hornick, Andrew J. Wagner

**Affiliations:** 1 Ludwig Center at Dana-Farber/Harvard and Center for Sarcoma and Bone Oncology, Department of Medical Oncology, Harvard Medical School, Boston, MA USA; 2 Department of Orthopaedic Surgery, School of Medicine, Keio University, Tokyo, Japan; 3 Department of Radiology, Brigham and Women’s Hospital, Harvard Medical School, Boston, MA USA; 4 Department of Radiology, University Hospitals Cleveland Medical Center, Case Western Reserve University, Cleveland, OH USA; 5 Department of Orthopedic Surgery, Brigham and Women’s Hospital, Harvard Medical School, Boston, MA USA; 6 Department of Surgery, Brigham and Women’s Hospital, Harvard Medical School, Boston, MA USA; 7 Department of Pathology, Brigham and Women’s Hospital, Harvard Medical School, Boston, MA USA

**Keywords:** Tenosynovial giant cell tumor, Malignant tenosynovial giant cell tumor, Malignant transformation, Soft tissue sarcoma, Metastasis, Chemotherapy, Tyrosine kinase inhibitors

## Abstract

**Background:**

Although tenosynovial giant cell tumor (TGCT) is classified as a benign tumor, it may undergo malignant transformation and metastasize in extremely rare occasions. High aberrant expression of CSF1 has been implicated in the development of TGCT and recent studies have shown promising activity of several CSF1R inhibitors against benign diffuse-type TGCT; however, little is known about their effects in malignant TGCT.

**Case presentation:**

Information from six consenting patients (3 men, 3 women) with malignant TGCT presenting to Dana-Farber Cancer Institute for initial or subsequent consultation was collected. Median age at initial diagnosis of TGCT was 49.5 years (range 12–55), and median age at diagnosis of malignant TGCT was 50 years (range 34–55). Two patients developed malignant TGCT de novo, while four other cases showed metachronous malignant transformation. All tumors arose in the lower extremities (3 knee, 2 thigh, 1 hip). Five patients underwent surgery for the primary tumors, and four developed local recurrence. All six patients developed lung metastases, and four of five evaluable tumors developed inguinal and pelvic lymph node metastases. All six patients received systemic therapy. Five patients were treated with at least one tyrosine kinase inhibitor with inhibitory activity against CSF1R; however, only one patient showed clinical benefit (SD or PR). Five patients were treated with conventional cytotoxic agents. Doxorubicin-based treatment showed clinical benefit in all four evaluable patients, and gemcitabine/docetaxel showed clinical benefit in two patients. All six patients died of disease after a median of 21.5 months from diagnosis of malignant TGCT.

**Conclusions:**

This study confirms that TGCT may transform into an aggressive malignant tumor. Lymph node and pulmonary metastases are common. Local recurrence rates are exceedingly high. Conventional cytotoxic chemotherapy showed clinical benefit, whereas tyrosine kinase inhibitors against CSF1R showed limited activity. Given its rarity, a prospective registry of malignant TGCT patients is needed to further understand the entity and to develop effective strategies for systemic treatment.

## Background

Tenosynovial giant cell tumor (TGCT), previously termed pigmented villonodular synovitis, was initially described by Jaffe et al. in 1941, and is currently categorized as a fibrohistiocytic tumor according to the WHO classification [1, 2]. TGCTs are subclassified based on growth patterns (localized- and diffuse-types) as well as on the location (tendon sheath, and intra- and extra-articular forms). Diffuse-type TGCT is clinically characterized by infiltrative growth and is notorious for its high local recurrence rate [2–4]. Histologically, mononuclear synovial-like neoplastic cells constitute a smaller portion of each tumor and are admixed with abundant infiltrates of multinucleated osteoclast-like giant cells and other inflammatory cells. Recent studies have demonstrated that the neoplastic cells contain a t (1;2) (p13;q37) translocation that results in a *COL6A3-CSF1* fusion gene and dysregulated expression of CSF1 RNA and protein in the majority of cases [5–7]. A novel fusion gene involving the CSF1 gene was also identified in another study [8]. This high expression of aberrant CSF1 is currently implicated in causing autocrine and paracrine signaling and a large inflammatory cell component in TGCT.

Although diffuse-type TGCT is classified as a benign tumor, in extremely rare occasions it may metastasize. Thirty cases of malignant TGCT have been reported in the English literature to date, showing its aggressive nature and significant risk of mortality (33–50%) [4, 9, 10], while a few case reports have shown cases of dissemination of benign TGCT after surgical interventions [11, 12]. Several molecularly targeted drugs have inhibitory activity against CSF1R, the receptor of CSF1, and suppress its downstream signaling. Although recent studies have shown promising activity of kinase inhibitors against benign diffuse-type TGCT [13–18], little is known about their effects in the malignant form of the disease. In this study we report the pathologic, clinical, and imaging characteristics as well as clinical outcomes following systemic treatment of patients with TGCT with evidence of malignancy.

## Case presentation

Patients prospectively provided signed informed consent for an Institutional Review Board-approved protocol for research use of medical records, of pathologic specimens obtained as part of routine clinical care, and publication. Information was collected from the medical records of all consenting patients with malignant TGCT presenting to Dana-Farber Cancer Institute for initial or subsequent consultation. Pathology slides were reviewed by a pathologist (JLH), and imaging features and patterns of response to treatment were evaluated by a radiologist (JPJ) with clinical expertise in soft tissue sarcomas. The clinical history for one patient (case 3) was previously reported in a series of patients with TGCT treated with imatinib [14], and imaging characteristics were recently described [19]. Since 2005, six patients with TGCT with clear histological evidence of malignancy have been treated at our institute.

### Case 1

**Fig. 1:**
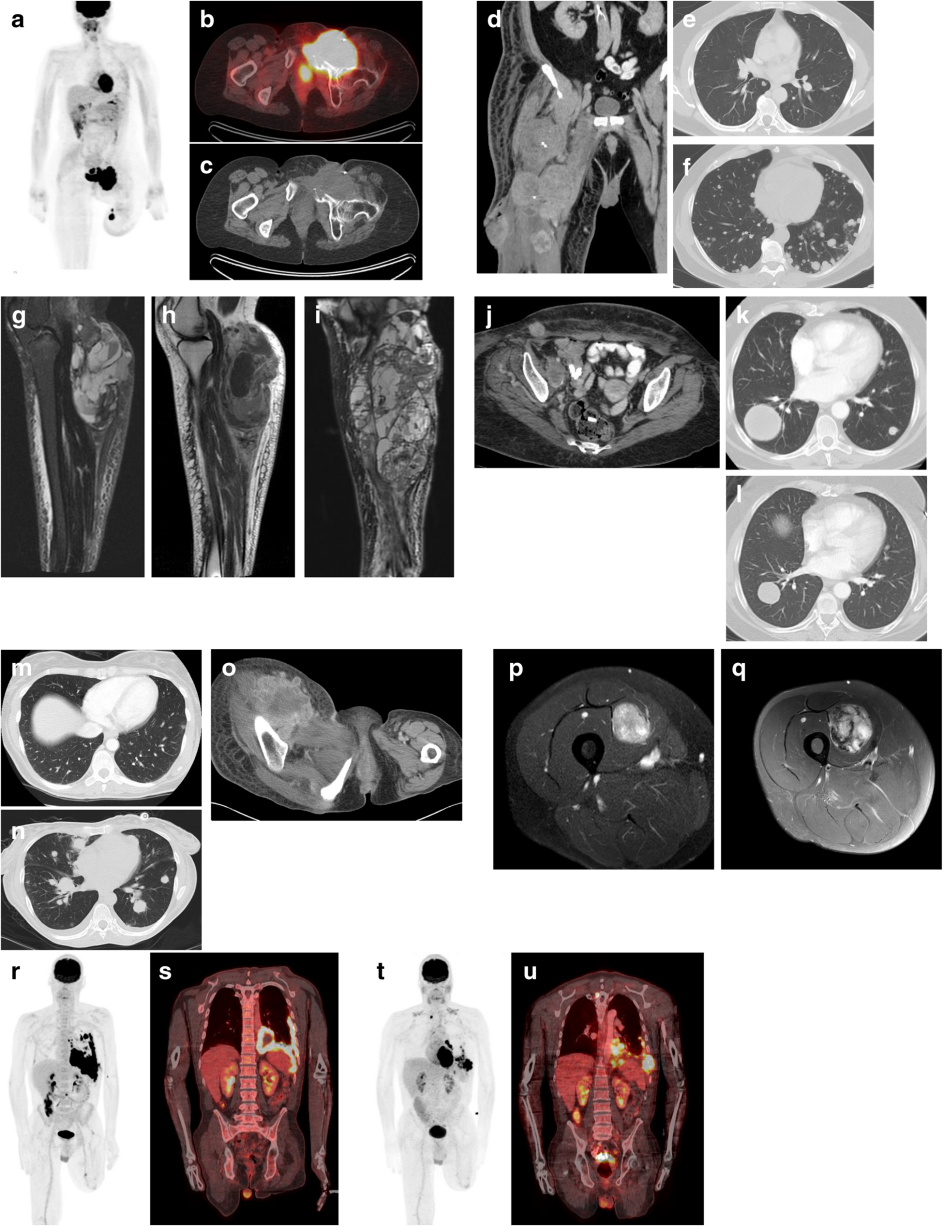
**a, b, and c**. (Case 1) PET-CT demonstrates a huge multilobulated mass centered in the left anterior pelvis at the left external iliac nodal region with destruction of left superior pubic ramus and additional FDG avid nodules in the left proximal medial thigh close to the amputation stump . D-F. (Case 2) Coronal contrast enhanced CT of the pelvis and lower extremity (**d**) reveals multifocal soft tissue masses in the right thigh. One of the lateral thigh mass has fistulized to the skin surface. Axial contrast enhanced CT of the chest (**e**) showed scattered small pulmonary nodules. Follow up chest CT after treatment with sorafenib showed dramatic increase in size and number of pulmonary nodules (**f**). G-L (Case 3). Sagittal STIR and post-contrast T1 W MR images of the right leg at presentation shows a large T2 heterogenous mass with multiple fluid –fluid levels and heterogenous enhancement in the posterior calf and leg (**g, h**). Sagittal STIR MR images three months following resection demonstrates a large complex T2 heterogenous enhancing mass within the resection site consistent with recurrent tenosynovial giant cell tumor (**i**). Axial contrast enhanced CT of the pelvis (**j**) and the chest (**k**) showed right inguinal and pelvic lymph node metastases and bilateral pulmonary metastases. Chest CT following four cycles of doxorubicin/ifosfamide showed interval improvement with decrease in size of the dominant right lower lobe mass and near complete resolution of the smaller pulmonary nodules (**l**). M-O (Case 4). CT images before (**m**) and after (**n, o**) nilotinib treatment showing tumor progression in the lung and in the right thigh. P-Q (Case 5). Axial post-contrast T1 W MR images at presentation shows well circumscribed mildly lobulated intramuscular mass in the vastus medialis muscle with nearly homogenous contrast enhancement (**p**). Axial post-contrast T1 W MR images 3 years later, showed interval increase in size of the mass with new heterogenous enhancement (**q**). R-U (Case 6). Coronal MIP, Coronal and axial fused PET-CT images demonstrates intensely FDG avid pleural metastases in the left hemithorax (**r, s**). Follow up images after treatment with imatinib shows mixed interval response with decrease in size and avidity of some of the pleural tumor and new rib destruction and chest wall extension (**t, u**)

A 10-year-old girl first noted a swollen left knee and underwent repeated arthrocentesis. At age 13, she underwent arthroscopic surgery and was diagnosed with benign TGCT. She subsequently underwent numerous synovectomies to treat local recurrences and radiation therapy to her left knee joint at age 15. Ultimately, her disease spread to her upper calf and posterior thigh. At age 32, her upper calf lesion was resected, and the tumor in her posterior thigh was treated with radiation. At age 34, swelling of an inguinal lymph node was noted and fine needle aspiration was consistent with malignant TGCT. Other staging scans revealed a pelvic mass and a sub-centimeter pulmonary nodule. Her disease remained stable after four cycles of doxorubicin/ifosfamide and she subsequently received gemcitabine/docetaxel as well as radiation therapy to her pelvis with stable disease for three months. She underwent left-sided above the knee amputation and excision of the intrapelvic masses. Eight months later, enlarging pulmonary nodules were resected. She was treated with sirolimus (rapamycin) and remained disease-free for eight months until a pulmonary nodule and two inguinal masses were noted. In 2007, at age 37, she was referred to our hospital and treated with sorafenib for four months with mixed response. She subsequently began on sunitinib 37.5 mg daily. Interval restaging scans showed no evidence of progressive disease for 15 months. She had significant interval progression of a right-sided pelvic mass while she temporarily stopped sunitinib in the perioperative setting of resection of painful metastatic nodules near the amputation stump (Fig. 1a, b, and c). Given that the drug had a significant role of controlling the disease, her sunitinib was restarted in combination with sirolimus (rapamycin). Her disease was under good control for eight additional months until she had progressive disease in the pelvis and underwent hemipelvectomy. She died of the disease at age of 39, six years after the malignant transformation.

### Case 2

A 53-year-old man was diagnosed with malignant TGCT after undergoing resection of a soft tissue tumor in the right proximal rectus femoris muscle. He underwent adjuvant radiation therapy but developed a local recurrence and underwent another wide resection five months after the initial wide resection. One year after the initial wide resection, he developed further progression of his tumor in the thigh and repeat staging evaluation revealed involvement of a right inguinal lymph node and bilateral small pulmonary nodules (Fig. 1d, and e). He was enrolled in a clinical study with sorafenib (NCT00330421); however, he was taken off study after a month because of dramatic disease progression in the lungs (Fig. 1f) as well as in the thigh. The patient died of disease 20 months after diagnosis.

### Case 3

A 55-year old woman presented with an enlarging mass in the posterior aspect of her right knee. The initial MRI revealed an extensive soft tissue lesion in the calf with fluid levels present (Fig. 1 g, and h). Biopsy was consistent with a benign diffuse-type TGCT. Three months after she underwent resection of the mass in the posterior popliteal fossa, she developed a local recurrence (Fig. 1i). Above-knee amputation followed the second resection of the recurrent tumor whose pathology revealed malignant transformation. CT scans two months later revealed metastases to the right inguinal nodes and the lung (Fig. 1j, and k). She subsequently began imatinib, but treatment was terminated because of progressive disease. Four cycles of doxorubicin/ifosfamide demonstrated partial response, resulting in disappearance of pulmonary nodules and significant decrease in size of inguinal nodules (Fig. 1l). She was enrolled in two clinical trials of investigational mTOR inhibitor or placebo and of an investigational PI3K/mTOR inhibitor, neither of which provided significant clinical benefit by the time of first follow-up CT. She then completed two cycles of liposomal doxorubicin, with no clinical benefit. She was subsequently treated with two cycles of ifosfamide and palliative radiation to her leg, resulting in marked response of the tumor in the thigh, but progressive disease systemically. Her disease then showed partial response to gemcitabine/docetaxel for five months before developing progressive disease in the lungs. She died of the disease 23 months after the diagnosis of malignant TGCT.

### Case 4

A 46-year-old woman presented with a 10-cm mass involving the right psoas muscle, the gluteal muscles, and the iliac bone. A CT scan at presentation demonstrated sub-centimeter pulmonary nodules, in addition to the pelvic mass. The CT-guided biopsy was consistent with malignant TGCT. Cytogenetic studies demonstrated an unbalanced t (1;2) translocation. Because of severe pain in her right hip, she underwent palliative radiation therapy (54 Gy) to her right pelvis, which dramatically improved her intractable pain. Her disease remained stable after four cycles of doxorubicin/ifosfamide. She enrolled in a clinical trial of nilotinib for TGCT (NCT01207492) but progressed after 1 month. She was then treated with gemcitabine/docetaxel without clinical benefit, and she died of the disease nine months after diagnosis.

### Case 5

A 44-year-old man first noted a mass in the vastus medialis. A biopsy demonstrated a diagnosis of benign diffuse-type TGCT (Fig. 1p). The patient elected to follow a course of observation. Three years later, he noted an increase in the size of the mass and subsequently underwent a marginal excision of the tumor. Pathology was consistent with malignant TGCT with positive margins (Fig. 1q). Staging CT revealed multiple pulmonary metastases. The patient subsequently participated in a blinded clinical trial of doxorubicin with an investigational drug or placebo and developed a partial response; he completed 6 cycles before electing to stop chemotherapy. He later developed metastatic disease to the subcutaneous tissue, pleura, liver, mesentery, bones, and sacral nerve roots and was treated with palliative radiation to the spine with concurrent paclitaxel, which failed to control his disease. Subsequent gemcitabine/vinorelbine provided no clinical benefit, and he died of disease 17 months after diagnosis of malignant TGCT.

### Case 6

A 54-year-old man noted progressive discomfort and swelling in his left knee. Resection of the lesion revealed benign diffuse-type TGCT. Despite resection, he developed rapid recurrence within weeks. Given the aggressive nature of the recurrent tumor, the patient was treated with two cycles of doxorubicin/ifosfamide in a neoadjuvant setting that was stopped for progression of disease, and he then underwent above-knee amputation. Pathology confirmed multifocal malignant transformation of TGCT. One year after amputation, he developed painless inguinal lymphadenopathy from metastatic TGCT. Six months later he developed malaise, dyspnea on exertion, nonproductive cough, and night sweats. Chest CT demonstrated a left sided pleural mass which was confirmed as metastatic malignant TGCT by biopsy. PET-CT revealed several pulmonary nodules, a focal hypermetabolic lesion in the L3 vertebral body, and a rapidly progressing pleural lesion. Systemic treatment with imatinib showed mixed response, with resolution of FDG avidity of the bulk of the mass but development a new hypermetabolic component invading his chest wall. He received palliative radiation to this mass and then initiated pazopanib but within one week developed symptomatic progression and entered hospice care. He died 3 years after initial diagnosis, 2 years after diagnosis of malignant TGCT, and 13 months after he developed metastatic disease.

### Clinical presentation and pathologic findings

We treated six patients (three men, three women) with median age at initial diagnosis of TGCT of 49.5 years (range 12–55) and median age at diagnosis of malignant TGCT of 50 years (range 33–55) (Table 1). Two patients presented malignant TGCT de novo, with histologically confirmed malignant findings in the primary tumors (Cases 2 and 4), while four other cases showed metachronous malignant transformation after previous diagnosis of a benign tumor. Conventional TGCT is composed of a polymorphous population of cells, including osteoclast-like giant cells, lymphocytes, foamy histiocytes, small mononuclear cells (histiocytes), and larger, cytologically benign mononuclear cells with an epithelioid appearance, eccentric nuclei, and eosinophilic cytoplasm; small histiocytes are often the dominant cell population, whereas the larger mononuclear cells (the neoplastic cellular component) usually represent only a minor contribution to the infiltrate. In contrast, malignant TGCT is usually dominated by large mononuclear epithelioid cells with marked nuclear atypia; some examples resemble an undifferentiated pleomorphic sarcoma. Proper diagnosis of malignant TGCT requires identification of a conventional (benign) component or a prior history of a conventional TGCT. Immunohistochemistry plays a limited role in the diagnosis of both conventional and malignant TGCT. One of the metachronous malignant TGCT was associated with prior radiation to a repeatedly recurrent benign tumor 21 years after the initial diagnosis of benign TGCT (Case 1). Two of the metachronous cases (Cases 3 and 6) developed local recurrences in malignant forms within three months. All tumors originated in the lower extremities, three in the knee, two in the thigh, and one in the hip. Two tumors showed clear evidence of intra-articular origins (Cases 1 and 6).

**Table 1:** Patients’ demographics

Case	Sex	Primary site	Age (Y)		de novo or metachronous (lag time)	Surgery	Adjuvant radiotherapy	Local recurrence	Additional surgery	Lymph node involvement	Pulmonary metastasis
			At initial diagnosis	At diagnosis with mTGCT							
1	Female	Knee joint	12	33	metachronous (21 years)	Amputation	No	Yes	Hemipelvectomy	Yes	Yes
2	Male	Thigh	53	53	de novo	Resection	Yes	Yes	No	Yes	Yes
3	Female	Knee	55	55	metachronous (3 months)	Amputation	No	Yes	No	Yes	Yes
4	Female	Hip	46	46	de novo	Not indicated	Yes	.	.	N.A.	Yes
5	Male	Thigh	44	47	metachronous (3 years)	Resection	No	Yes	No	No	Yes
6	Male	Knee joint	55	55	metachronous (3 months)	Amputation	No	No	.	Yes	Yes

*mTGCT* malignant tenosynovial giant cell tumor

### Local treatment, local recurrence and distant metastasis

Surgery for the primary tumors was carried out in five cases, while it was not indicated for Case 4 due to her advanced primary pelvic tumor that invaded the psoas muscle, the gluteal muscles and the iliac bone (Table 1). Although three patients with knee primaries subsequently underwent above-knee amputation due to the involvement of the neurovascular bundle, two of them eventually developed local recurrence on their stumps (Cases 1 and 3), and additional hemipelvectomy was required for disease control in Case 1. Two patients who underwent surgery received adjuvant radiation therapy. Collectively, four of five patients developed local recurrence after surgery for the primary tumors. Case 4 was treated with radiotherapy as a palliative local treatment which resulted in improvement of pain for six months, and Case 6 also received palliative radiotherapy for pain control but died too soon afterwards to determine if this provided any benefit. All six patients developed metastases to the lung, and four of five evaluable patients showed metastases to the inguinal and pelvic lymph nodes (Table 1).

### Systemic therapy

All six patients received systemic therapy (Table 2). Five patients had at least one tyrosine kinase inhibitor (TKI) with described inhibitory activity against CSF1R. The TKIs used in this case series included sorafenib [20], sunitinib [21], imatinib [22–24], nilotinib [25, 26], and pazopanib [27–29]. Five patients had conventional cytotoxic agents, including doxorubicin/ifosfamide, doxorubicin and an investigational agent or placebo, and gemcitabine/docetaxel. The response to treatments in each case is shown in Table 2. Doxorubicin-based treatment showed clinical benefit (SD and PR) in all four evaluable patients, and gemcitabine-based treatment showed clinical benefit in two of four patients, while, among all TKIs used in this study, only sunitinib in Case 1 showed clinical benefit. All patients died of disease at 9, 17, 20, 23, 25 and 72 months (median: 21.5 months) from diagnosis of malignant TGCT.

**Table 2:** Systemic therapies and oncological outcomes

Case	Systemic therapy	Best response (time to progression)	Oncological outcomes	Time from diagnosis of malignant TGCT
1	Doxorubicin + ifosfamide Gemcitabine + docetaxel Sirolimus (rapamycin) Sorafenib Sunitinib alone Sunitinib + sirolimus	SD (4 M) SD (3 M) N.A. (8 M) PD (mixed response) SD (15 M) PR (8 M)	DOD	72 M
2	Sorafenib	PD	DOD	20 M
3	Imatinib Doxorubicin + ifosfamide mTOR inhibitor PI3K/MTOR inhibitor Liposomal doxorubicin Ifosfamide Gemcitabine + docetaxel	PD PR (4 M) PD PD PD PD PR (5 M)	DOD	23 M
4	Doxorubicin + ifosfamide Nilotinib Gemcitabine + docetaxel	SD (3 M) PD PD	DOD	9 M
5	Doxorubicin (+/− an investigational drug) Paclitaxel Gemcitabine + vinorelbine	PR (6 M) PD PD	DOD	17 M
6	Doxorubicin + ifosfamide Imatinib Pazopanib	N.A. PD (mixed response) Too short to evaluate(1 W)	DOD	25 M

*SD* stable disease, *N.A* data not available, *PD* progressive disease, *PR* partial response, *DOD* dead of disease; and *AWD* alive with disease

## Discussion and conclusions

This report provides evidence that TGCT has the potential to transform into an aggressive malignant tumor, although this is an exceedingly uncommon event. The annual incidence of benign (typical) TGCT is estimated to be 1.8 patients per million population in the USA [30]. Based on the number of cases in the current study (*n* = 6) and the previous report in which Li et al. reviewed 30 cases of malignant TGCT in the literature from 1979 [10], the incidence of malignant TGCT can be roughly estimated to be less than 0.1% of benign TGCT, with caveats of reporting biases and challenges in pathologic diagnosis. It is notable that roughly half of the malignant TGCT cases developed in a metachronous manner. Even though it is an extremely rare event, physicians need to be aware of the existence of this entity and to consider malignant transformation if a residual tumor or a recurrent tumor progresses rapidly. It is also notable that four of five evaluable tumors metastasized to the inguinal and pelvic lymph nodes (Table 1), similar to previous reports [10]. There is a very strong referral bias in the cases included here, wherein patients were referred to a medical oncologist in a Sarcoma Center because of the development of malignant disease, although 4 of the patients lived in the region. Statistically, transformation is an exceptionally rare event and we do not recommend routine staging or surveillance imaging outside of the affected areas unless there are unusual histologic features or the onset of new symptoms concerning for malignancy.

### Local treatment

With regards to the local treatment for the case of benign TGCT, surgery is the mainstay of the treatment. Achieving the proper balance between local control and function preservation for a locally aggressive benign tumor is challenging, because the tumor could at times recur after radical resection and at others not recur or remain indolent despite incomplete resection. A conservative approach is becoming more appropriate as new medical therapies are under development for benign TGCT (see below). However, once the localized tumor transforms to a malignant form, an early decision of radical resection should be considered in order to prevent further progression of disease, similar to management of other high-grade sarcomas. It is notable that three of five patients with malignant TGCT cases of the knee eventually underwent above-knee amputation and two of them had local recurrences on their stump. We were not able to define the clinical role of radiation therapy in malignant TGCT in this study due to the small sample size, but two patients showed demonstrable symptomatic improvement after palliative radiotherapy (Cases 3 and 4).

### Systemic therapy

Since the identification of aberrant CSF1 expression in TGCT, drugs that inhibit the CSF1/CSF1R signaling pathways have been tested in this disease. [13–18]. Blay et al. initially reported a patient with benign TGCT of the elbow that showed a complete response following treatment with imatinib, a TKI known to have activity against ABL, KIT, and PDGFR, but also with CSF1R inhibitory activity [23, 24], providing proof of concept for targeting CSF1R in TGCT [13]. Cassier et al. subsequently reported on 29 patients with TGCT treated with imatinib, including 2 patients with malignant disease (one of which is patient 3 in our cohort). Treatment with imatinib led to 19% partial responses and stable disease in 74% based on RECIST [14]. The high rate of patients who showed stable disease (74%) could suggest a mainly cytostatic effect of imatinib in TGCT although this may also reflect an often indolent behavior of this disease. Neither of the two patients with malignant disease in the Cassier report had clinical benefit from imatinib. Parallel Phase II studies of nilotinib, another drug with CSF1R inhibitory activity, in patients with advanced, recurrent and/or inoperable TGCT have been conducted in Europe (NCT01261429) and the US (NCT01207492) with the 12-week PFR of 85.7% (95%CI, 57.2–98.2%) from the European study, in favor of the study continuation. More recently, the effects of pexidartinib, a novel oral TKI with potent activity against CSF1R, have been reported. In the expansion cohort of a Phase 1 study, 23 patients with TGCT were enrolled and 12 (52%) had a partial response by RECIST and 7 had stable disease [17]. No patients with malignant disease were included in this published cohort. Substantial improvement in overall joint functionality, as well as decreased pain and stiffness, was also noted. In addition to small molecule kinase inhibitors, antibodies against CSF1R such emactuzumab (RG7155), have also been developed. In a Phase I study, 29 patients with advanced TGCT were enrolled, and 24 of 28 (86%) evaluable patients achieved an objective response, which was associated with good functional outcome [15]. No patients with malignant disease were included in this cohort. Although these studies have shown promising activities of recently developed CSF1R targeted drugs in benign TGCT cases, little have known about their activity in malignant forms of the disease.

Five patients in our report were treated with a variety of TKIs (Table 2) but only one obtained sustained clinical benefit (Case 1; stable disease for 23 months on sunitinib). It is not clear whether this agent, which also inhibits the vascular endothelial growth factor receptor and many other kinases, has particular activity against malignant TGCT, or if there were particular characteristics of this patient’s disease which made it more susceptible. Of note, this patient’s disease presented as typical TGCT and had a lengthy indolent course until malignant transformation and metastases later developed. Given the reported activity of pexidartinib and emactuzumab in benign TGCT cases, agents such as these that more potently inhibit the CSF1R pathway are of particular interest in the management of malignant TGCT. However, although CSF1R signaling appears to be critically important for benign TGCT, its role and that of other signaling pathways in malignant disease remains to be determined.

While TKIs had very limited activity in malignant TGCT in our series, clinical benefit was observed in three of four patients who received anthracycline-based therapy and two of three who received gemcitabine/docetaxel. The effects of potent CSF1R inhibitors should still be explored in the management of malignant TGCT, but conventional cytotoxic chemotherapy may also be considered as part of the treatment strategy of metastatic disease.

Despite the small sample size, this study provides valuable clinical data that demonstrates extremely aggressive nature of malignant TGCT which frequently recur locally, metastasize to the lymph nodes and to the lung, and all six patients died of disease after a median of 21.5 months from diagnosis. With regards to systemic therapy, conventional cytotoxic chemotherapy including anthracycline-based therapy and gemcitabine/docetaxel showed clinical benefit, whereas molecular targeted therapy targeting the CSF1/CSF1R pathway showed limited activity. Given its rarity, a prospective registry of malignant TGCT patients is needed to further understand the entity and to develop effective strategies for systemic treatment.

## Abbreviations

DOD: Dead of disease; and AWD, alive with disease.
mTGCT: Malignant tenosynovial giant cell tumor
N.A: Data not available
PD: Progressive disease
PR: Partial response
SD: Stable disease
TGCT: Tenosynovial giant cell tumor
TKI: Tyrosine kinase inhibitor
